# Ultrathin and Highly Conformal Self-Powered Sensors by Liquid-Phase Transferring

**DOI:** 10.34133/research.0785

**Published:** 2025-07-29

**Authors:** Xingyi Dai, Qihua Liang, Yinghui Wu, Jiaxin Han, Yajun Cao, Xuyang Zhang, Junhui Huang, Junle Qu, Long-Biao Huang, Jie Kong, Jianhua Hao

**Affiliations:** ^1^ Key Laboratory of Optoelectronic Devices and Systems of Ministry of Education and Guangdong Province, College of Physics and Optoelectronic Engineering, Shenzhen University, Shenzhen 518060, P. R. China.; ^2^ Department of Applied Physics, The Hong Kong Polytechnic University, Hong Kong, P. R. China.; ^3^ National Key Laboratory of Green and Long-Life Road Engineering in Extreme Environment, Shenzhen University, Shenzhen 518060, P. R. China.; ^4^ MOE Key Laboratory of Materials Physics and Chemistry in Extraordinary Conditions, Shaanxi Key Laboratory of Macromolecular Science and Technology, School of Chemistry and Chemical Engineering, Northwestern Polytechnical University, Xi’an 710072, P. R. China.

## Abstract

Self-powered sensing technologies have sparked a revolution in electric devices. Furthermore, ultrathin characteristics are highly desirable for on-skin and wearable devices to achieve superior conformability on complex 3-dimensional surfaces, which facilitates improved wearing comfort and detection accuracy. However, developing self-powered sensors with ultrathin and conformal features without complicated fabrication processes remains a formidable challenge. Herein, we present an ultrathin self-powered sensor with high conformability, fabricated by a liquid-phase transferring approach. The sandwich-like sensor is spin-coated layer by layer on a water-soluble substrate. Upon immersion in water and complete dissolution of the sacrificial layer, the sensor can be transferred to a variety of surfaces with diverse morphologies. The ultrathin sensor shows long-term stability. When the 45-μm-thick sensor is transferred to human skin, robotic hands, insole, flat plates with fine bevels, cylinders, undulating surfaces, and leaf textures, the fingerprint and surface details of the objects are vividly reflected on the sensor surface, attesting to its exceptional conformability. Driven by the triboelectric effect, the self-powered sensor and its array exhibit good sensitivity and rapid response time, enabling tactile sensing functions for pressure, material species, surface roughness detection, and motion state. The proposed design strategies for ultrathin self-powered sensors hold immense promises in wearable devices, robotics, and human–machine interfacing.

## Introduction

Wearable devices with intelligent and portable features facilitate a more convenient and personalized lifestyle for users, promote health management, and enhance work efficiency [1–3]. However, the prevalent high power consumption of numerous electronic devices poses a substantial concern in the contemporary energy landscape. Furthermore, the disposal of discarded batteries escalates the maintenance expenses of electronic equipment and engenders environmental pollution [4]. Self-powered sensing technologies offer a promising avenue for battery-free devices by harnessing natural energy sources to sustain device operation, thus mitigating the issue of short battery life and the need for frequent battery replacements [5–9].

Various energy sources, including mechanical, light, thermal, and moisture energy, can be converted into electricity by piezoelectric, triboelectric, photovoltaic, thermoelectric, and moisture-electric generation technologies [10–14]. Among these self-powered technologies, triboelectric nanogenerators (TENGs) exhibit notable potential by capturing diverse mechanical energy that can be transformed into electricity or electrical signals via triboelectrification and electrostatic induction mechanisms [15–17]. TENG-based systems hold promise across diverse domains, including biomedicine, human–machine interaction, environmental monitoring, and motion sensing [18–23]. Moreover, triboelectric sensors based on TENG offer distinct advantages, including versatility of material selection, compactness, flexibility, and low cost, rendering them particularly suitable for self-powered wearable devices [24–26].

The triboelectric sensor, designed for self-powered wearable device, can be strategically positioned on diverse body regions to capture mechanical energy arising from motion, thereby generating electrical signals for sensing and monitoring [27–29]. Skin-attachable triboelectric sensors play a key role in acquiring various information, which are required to be flexible, thin, lightweight, and adaptable to various contours [30,31]. When the triboelectric sensors are in contact with the human epidermis or objects with concave–convex surfaces, the adaptability of the sensors to the complex shapes can ensure the sensitivity and accuracy of the sensing signals and enhance user comfort. Consequently, there is a pressing need to explore key technologies for developing ultrathin self-powered sensors.

Triboelectric sensors are commonly structured with multiple layers comprising triboelectric film and electrode layers [32,33]. To achieve thin films, several methods have been developed, including cast-molding, spin-coating, spraying, blade coating, sputtering, electrospinning, and thermal evaporation [34–39]. The spin-coating method is favored for its marked advantages such as mild process conditions, simple operation, wide material compatibility, thickness controllability, and cost-effectiveness. Zhong et al. [40] presented a TENG-based pressure sensor containing an ultrathin polydimethylsiloxane (PDMS) layer, which was applied through spin-coating and then peeled off from the substrate after curing. Wong et al. [35] combined spin-coating, sputtering, and photolithography to obtain the ultrathin tattoo-like TENGs with layer-by-layer structures comprising PDMS, polyimide (PI), copper (Cu), PI, and liquid bandage (LB), which could be tightly attached to skin through the adhesive LB. However, it is difficult to completely and non-destructively peel off the ultrathin sensors from solid substrates. Additionally, the use of additional adhesives to attach the sensors on the target may result in attenuation of the electrical signal. Therefore, transferring techniques play an important role in achieving ultrathin sensors with high performance.

Typically, ultrathin devices are transferred from the native substrates to the target substrates through solid or liquid carriers [41]. Among these, the liquid-phase transferring method has attracted increasing interest due to its simplicity and versatility in achieving high conformability to diverse surfaces. For instance, Liang et al. [42] employed polyvinylpyrrolidone as a sacrificial layer to fabricate an ultrathin AgNW–PDMS electrode by using a water-assisted transfer method, which prevented it from loosening, detaching, and sliding across the interface during the transfer process. Le Borgne et al. [43] demonstrated the use of water-soluble polyvinyl alcohol (PVA) substrates to transfer patterned films onto various 3-dimensional (3D) objects via the water transfer process. Despite the achievements in attaining ultrathin structures and good shape-adaptability with these few-layer films, the challenges associated with simple controllable transfer and high conformability to complex 3D surfaces. These issues are particularly relevant for multilayer self-powered sensors with multifunctional sensing capabilities, which persist and necessitate further attention.

Herein, we introduce an ultrathin self-powered sensor fabricated using liquid-phase transferring technology, which is capable of exceptional conformity to complex 3D surfaces. The sensor is spin-coated layer by layer onto a substrate containing the water-soluble polymer. After the sensor is immersed in water, the sensor can be transferred and conform to various surfaces, such as human skin, robotic hands, insole, flat plates with fine bevels, cylinders, undulating surfaces, and leaf textures. The total thickness of the 3-layer sensor consisting of PDMS and graphene-containing carbon paste is 45 μm. Operating in single-electrode triboelectric mode, the sensor on different surfaces can generate electrical signals under the external force to achieve self-powered sensing and shows long-term stability. The material types and surface roughness can be detected by the sensor on the robotic hands. The ultrathin sensor on the insole can be used to monitor human motion state such as walking and running. The ultrathin sensor array can be designed in 2 × 2 pixels for pressure detection. This work offers promising avenues for future advancements in flexible, ultrathin, and wearable devices.

## Results

### Design and preparation of ultrathin sensors

**Fig. 1.**
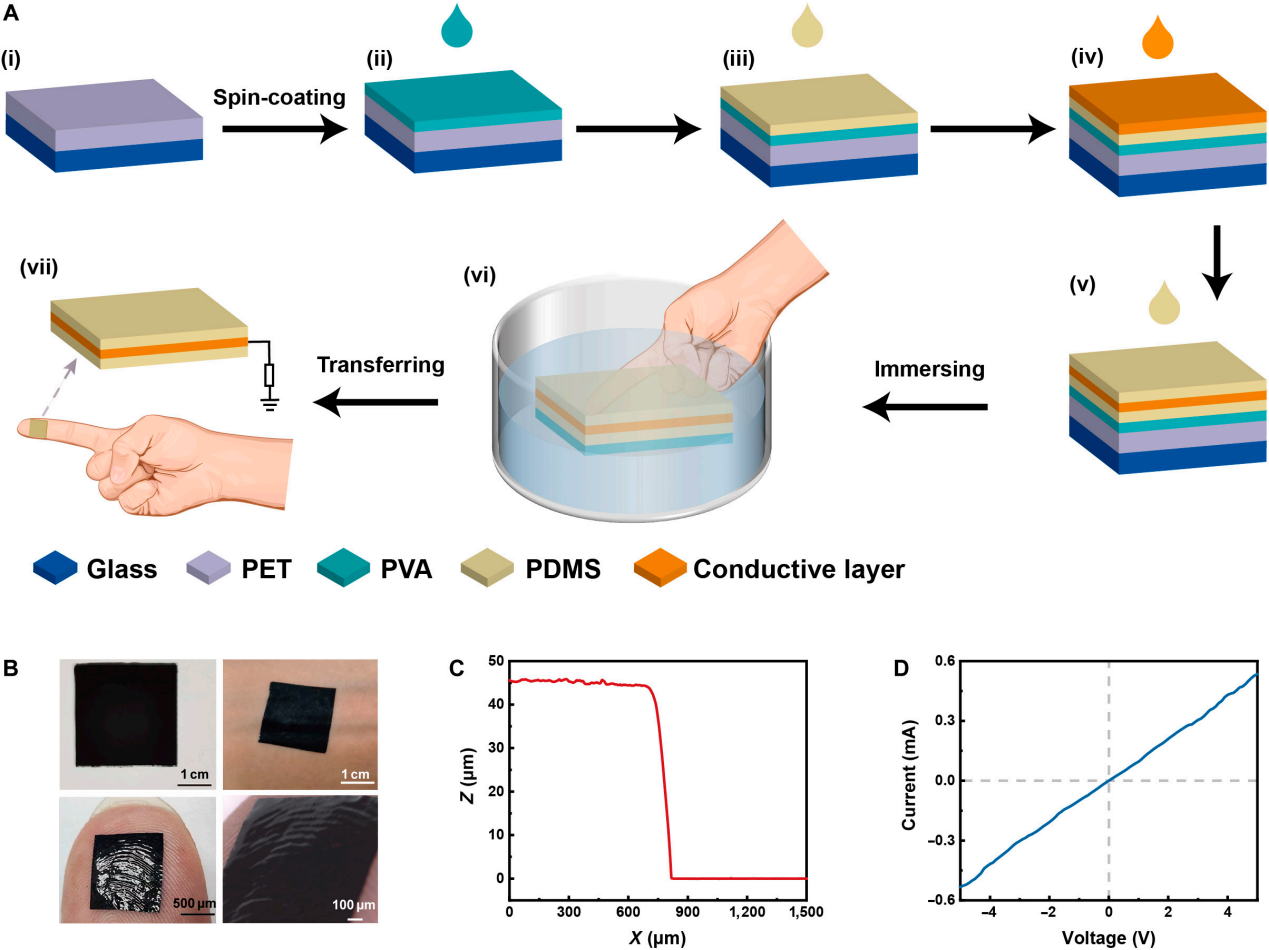
Preparation and characterization of the ultrathin self-powered sensor. (A) Schematic illustration of the preparation process of the ultrathin sensor via the liquid-phase transferring method. (B) Image of the ultrathin sensor attached to the skin. (C) Thickness of the ultrathin sensor with 2 PDMS layers and an electrode. (D) Current–voltage curves of the electrode layer of the sensor.

To achieve the ultrathin and highly conformal self-powered sensor for wearable devices, we introduce the liquid-transferring method. As shown in Fig. 1A, the preparation process of the ultrathin sensor involves a combination of spin-coating and liquid-phase transferring techniques. A polyethylene terephthalate (PET) film was adhered to a glass substrate. PVA, PDMS, graphene-containing carbon paste, and PDMS were spin-coated layer by layer. The device constructed by the multi-layer film was fully immersed in deionized (DI) water. After the PVA was completely dissolved, the device was detached from the PET film and was suspended in water. Upon contact with an object’s surface in water, the sensor could be transferred from the aqueous environment to the object’s surface. Once water evaporated, the sensor could be seamlessly affixed to the object. Eventually, the sandwich-like self-powered sensor was composed of 2 PDMS layers encapsulating a conductive electrode layer.

Due to the good electron affinity and nontoxicity, PDMS was selected to fabricate the triboelectric and supporting layer of the self-powered sensor. Graphene-containing carbon paste functioned as the conductive electrode. Additionally, a layer of water-soluble PVA, serving as a sacrificial layer, was spin-coated between the sensor and the PET film to facilitate the separation of the sensor from the PET substrate. In the spin-coating process, hydrodynamic modeling is influenced by parameters such as spin-coating time, angular velocity, fluid density, and viscosity. By adjusting these parameters, the final thickness of the film can be precisely controlled. In the transferring process, the liquid-phase transferring method enables the sensor with high conformability on object surfaces. During the transfer from water to the object’s surface, the air gap between the sensor and the object is filled with water molecules, and the sensor is tightly fitted to the object under the tension of the liquid surface. Subsequently, as water evaporates, the gap between the sensor and the object’s surface diminishes, and external atmospheric pressure facilitates the sensor securely attached to the object’s surface.

The ultrathin sensor fabricated by liquid-phase transferring can be conformably attached to human skin (Fig. 1B). Remarkably, benefiting from the flexible and ultrathin characteristics, the fingerprint of the thumb is visible on the sensor. The thickness of the sensor with a 3-layer structure is 45 μm (Fig. 1C). The resistance of the electrode, approximately 10 kΩ, can be calculated from the slope of the current–voltage curve (Fig. 1D). The as-fabricated sensor exhibited ultrathin performance and could well conform to human skin, making it possible for human sensing.

### Electrical performances of sensors

**Fig. 2.**
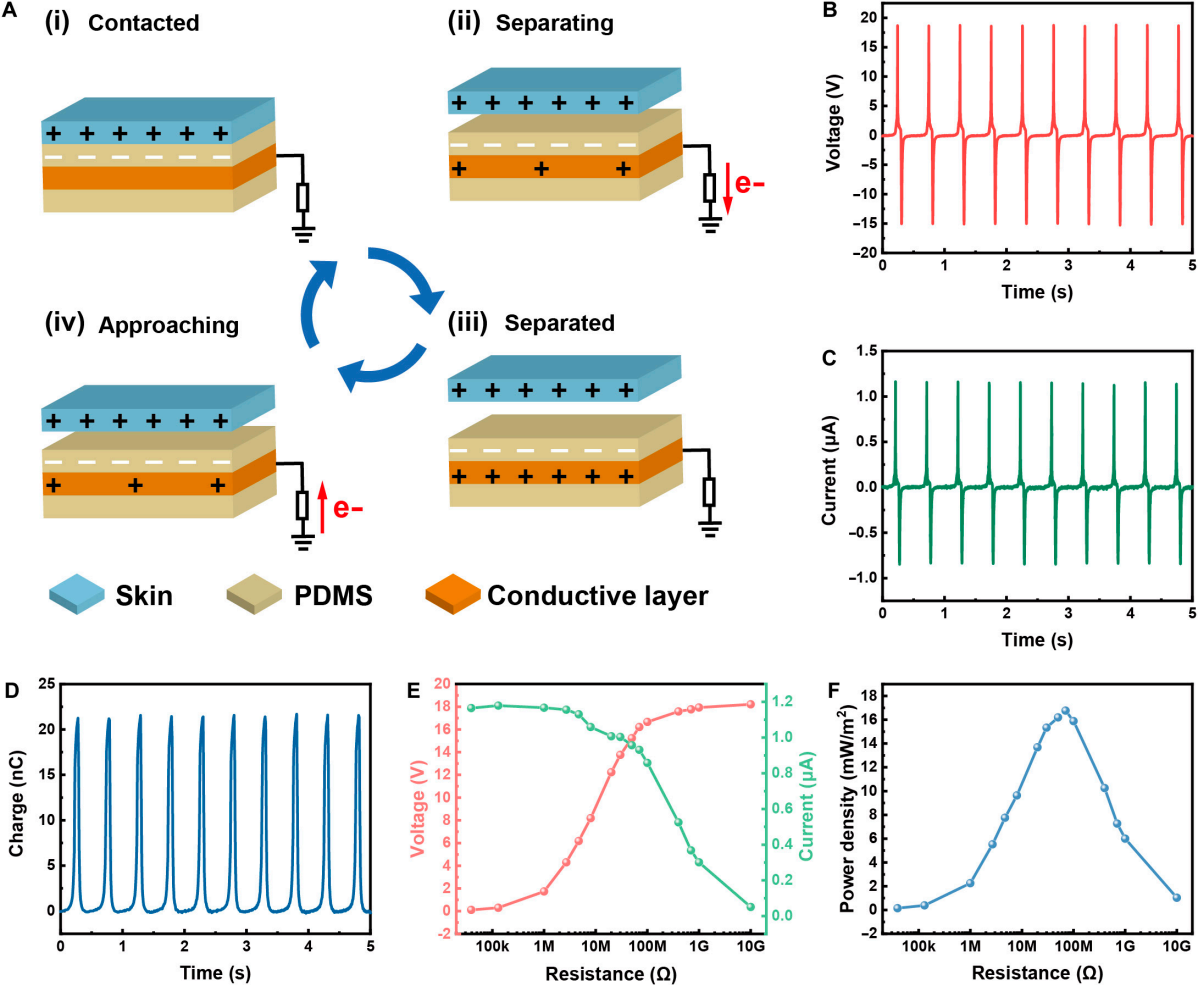
Ultrathin sensor based on triboelectric effect. (A) Working mechanism of the triboelectric sensor. (B) *V*_oc_, (C) *I*_sc_, and (D) *Q*_sc_ of the sensor during contact–separation at a frequency of 2 Hz. (E) Output voltage and current versus external load. (F) Power density versus resistance.

The self-powered sensor based on TENG could convert mechanical energy into electrical energy and signals during the contact–separation process, attributed to the synergistic effects of contact electrification and electrostatic induction. The working mechanism of the triboelectric sensor in the single-electrode mode is schematically depicted in Fig. 2A. PDMS and human skin differ in their ability to capture electrons, and electrons on the skin are captured by the PDMS surface when contact occurs. Equal amounts of positive and negative charges are distributed on the 2 surfaces, respectively. Owing to the equilibrium of electric potential, there is no flow of electrons from the external circuit (Fig. 2A-i). When the skin is lifted from the PDMS, the electrode layer is positively charged due to electrostatic induction, where the electric field formed by the negative charge on the PDMS drives the electrons to flow through the external load to ground (Fig. 2A-ii). After completing the separation process, the positive charge on the electrode layer is balanced with the electrons on the PDMS, resulting in no current flow (Fig. 2A-iii). When the skin and PDMS come back close together, the electrons at the ground end flow back into the electrode layer and there is a current in the opposite direction (Fig. 2A-iv). During the contact–separation cycle, a continuous alternating current (AC) is generated.

The sensor (30 mm × 30 mm) was transferred to the polymethyl methacrylate substrate to test the output electrical performances. When an external force with a frequency of 2 Hz is applied, the open-circuit voltage (*V*_oc_) is about 18.6 V (Fig. 2B). The short-circuit current (*I*_sc_) is 1.2 μA (Fig. 2C), and the short-circuit charge (*Q*_sc_) is about 22 nC (Fig. 2D). In the process of energy conversion, the internal circuit of TENG can be equated to a low-frequency AC voltage source in series with a capacitor, resulting in the large impedance. If the external load resistance is small, the circuit can be regarded as a short circuit due to the large internal impedance of TENG. At this point, the voltage is close to zero, while the current reaches maximum. When the resistance of the load circuit reaches above 10^9^ Ω, which is much larger than that of the TENG, the external circuit is equivalent to an open-circuit state. Therefore, the voltage across the external load is maximized, while the current tends to zero. In both states, the TENG provides too little power to the load, and there is no optimal match between the TENG and the external load. When the external load is close to the internal resistance of the TENG, an optimal match is achieved, and the output power reaches the peak value. As the resistance increases, the voltage across the load gradually becomes higher, and the current tends to decrease, which is in accordance with Ohm’s law (Fig. 2E). The peak value of the power density is 18 mW m^−2^ at the external load of 80 MΩ, achieving an optimal match (Fig. 2F).

**Fig. 3.**
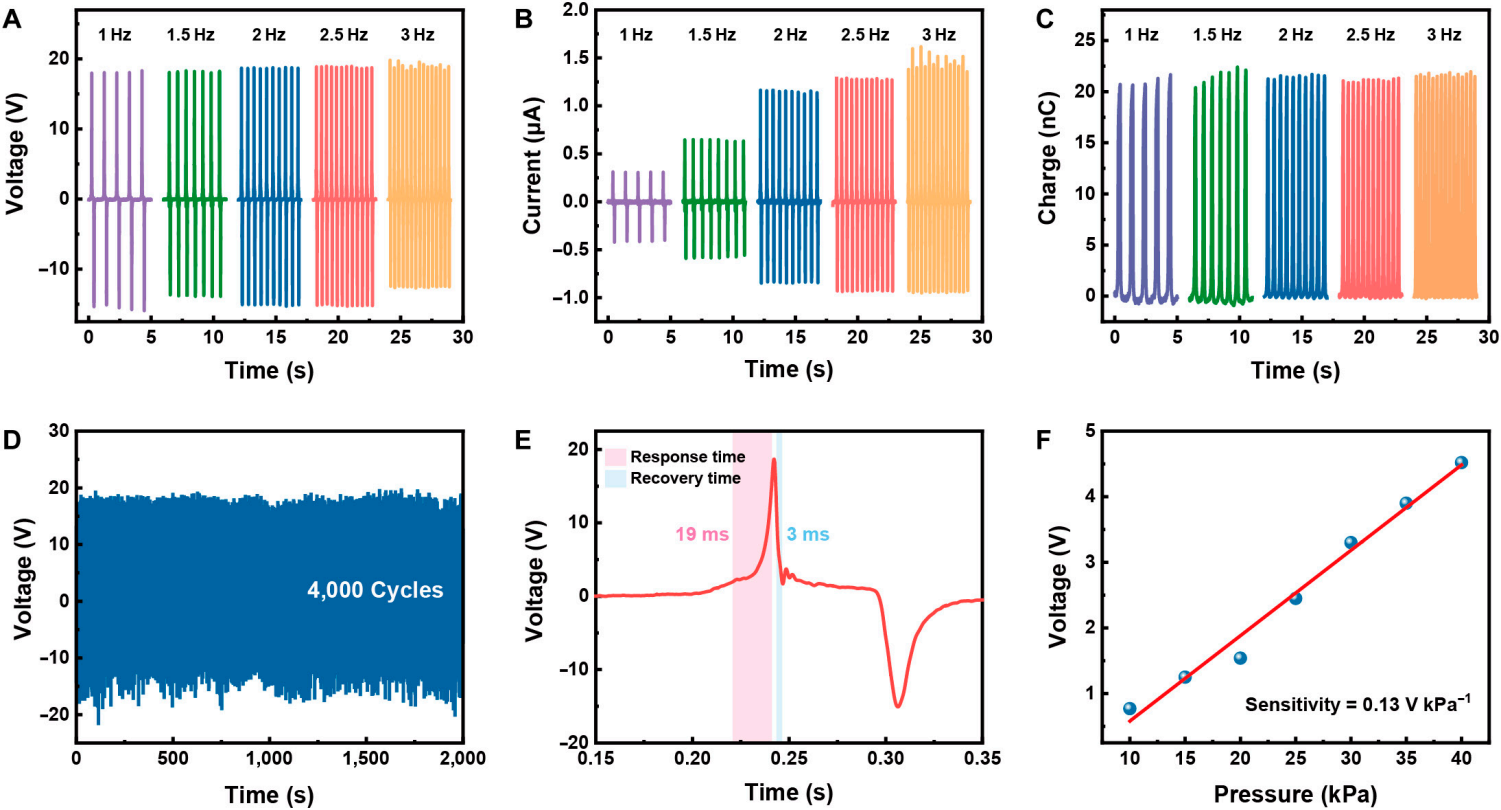
Electrical performance of the ultrathin triboelectric sensor. (A) *V*_oc_, (B) *I*_sc_, and (C) *Q*_sc_ of the sensor at frequencies ranging from 1 to 3 Hz. (D) Long-term stability of the output voltage over 4,000 cycles. (E) Response and recovery time of the sensor. (F) Output voltage versus pressure.

The electric performance of the ultrathin sensor related to frequency was investigated. As the frequency rises from 1 to 3 Hz, the *V*_oc_ and *Q*_sc_ almost remain unchanged, while the *I*_sc_ gradually increases (Fig. 3A to C). Since there was no dynamic process of charge transfer at the open-circuit condition, the frequency had little influence on the voltage. In addition, the voltage was affected by both the triboelectric charge density and the distance between the 2 triboelectric layers. Under the setting condition of contact electrification, the total charge transferred remained constant. However, the higher frequency accelerated the flow of electrons in the external circuit, leading to the increase of current [44]. The characteristic of stable voltage output at different frequencies is in favor of electrical sensing when the external forces are at irregular frequencies. Moreover, during the extended contact–separation process, the *V*_oc_ exhibits only minimal fluctuations over 4,000 cycles, indicating the good mechanical robustness and long-term stability of the sensor (Fig. 3D). The external force was applied using a digital force gauge to precisely measure the response time and sensitivity of the sensor. As illustrated in Fig. 3E, the sensor exhibits a response time of 19 ms, defined as the voltage rise from 10% to 90% of the peak voltage, and a recovery time of 3 ms, corresponding to the voltage drop from 90% to 10% of the peak voltage. These values demonstrate the sensor’s rapid response behavior, enabling the sensor to accurately detect signals promptly upon the application of external force. As shown in Fig. 3F, when the applied pressure increases, the output voltage correspondingly rises. An increase in pressure resulted in a larger effective contact area, thereby boosting the voltage response. Based on the proportional relationship between voltage and applied pressure, sensor sensitivity could be calculated by the slope of the fitting curves (voltage versus pressure). Between 10 and 40 kPa, the sensitivity is approximately 0.13 V kPa^−1^, validating the potential application of this sensor for pressure detection and wearable technologies.

### Conformability of sensors

**Fig. 4.**
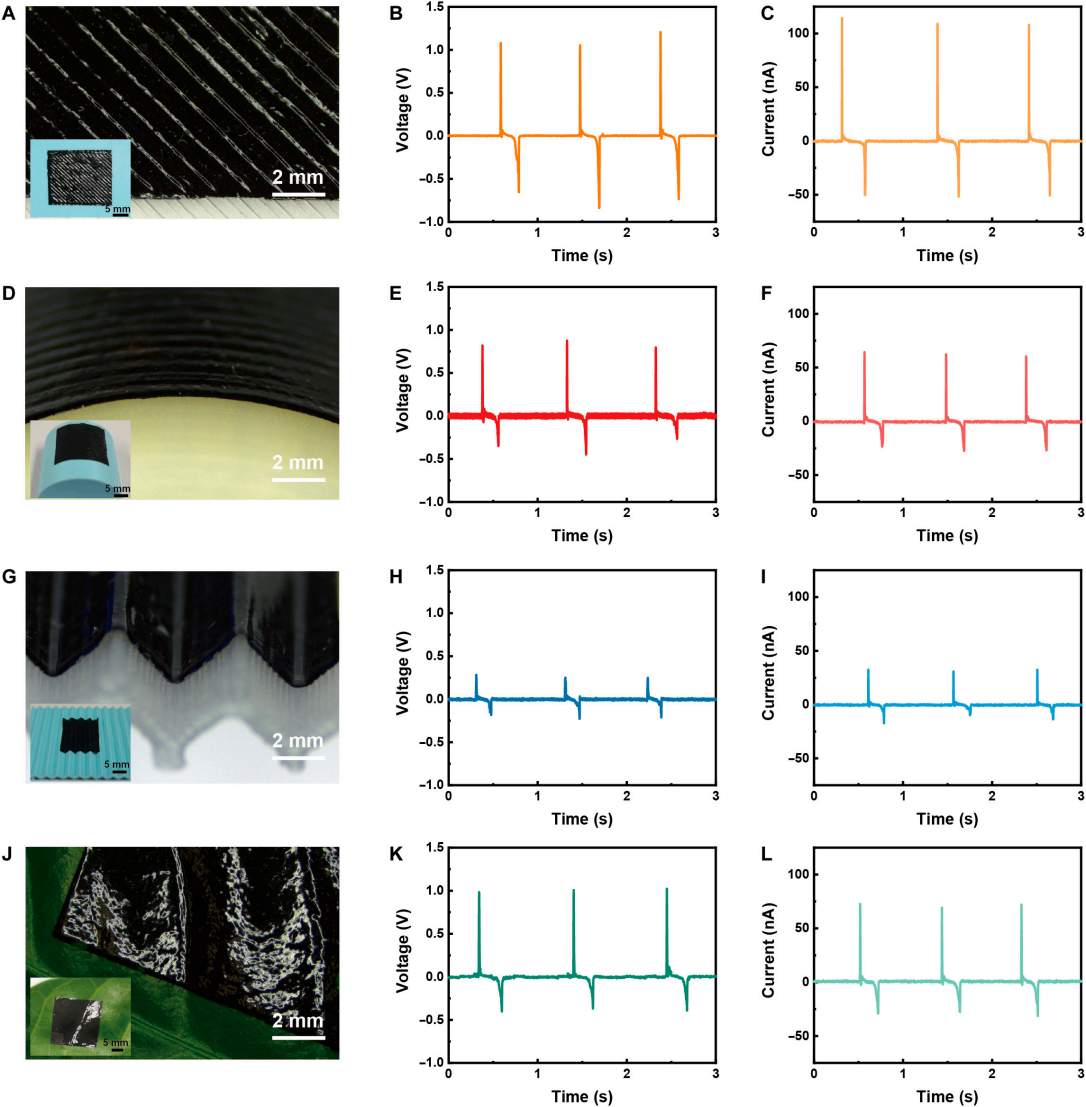
Conformability of the ultrathin sensor attached to different surfaces. (A) Image, (B) *V*_oc_, and (C) *I*_sc_ of the sensor attached to a flat plate with fine bevels. (D) Image, (E) *V*_oc_, and (F) *I*_sc_ of the sensor attached to a cylinder surface. (G) Image, (H) *V*_oc_, and (I) *I*_sc_ of the sensor attached to an undulating surface. (J) Image, (K) *V*_oc_, and (L) *I*_sc_ of the sensor attached to a leaf surface.

The ultrathin and flexible features are essential to provide electrical devices with conformability to form seamless contact on complex 3D surfaces. As shown in Fig. 4, through the liquid-phase transferring method, the as-fabricated sensor can be tightly attached to various surfaces, including flat plates with fine bevels (Fig. 4A), cylinder surface (Fig. 4D), undulating surfaces (Fig. 4G), and leaf surface (Fig. 4J). Surface stripes are visible on the device after the sensor is transferred to the object surface. The 3-layered sensor is thin and flexible, which makes it better for adhering to complex surfaces and different material surfaces. When touching the sensor on a flat surface with the index finger, the *V*_oc_ of 1.1 V and *I*_sc_ of 120 nA can be detected (Fig. 4B and C). The sensor on different surfaces generates different magnitudes of electrical signals. On a curved surface, the sensor generates the *V*_oc_ of 0.8 V and *I*_sc_ of 65 nA (Fig. 4E and F), while on an undulating surface, the *V*_oc_ of 0.3 V and *I*_sc_ of 30 nA can be generated (Fig. 4H and I). In addition, when the sensor is attached to a leaf, it can generate the *V*_oc_ of 1.0 V and *I*_sc_ of 75 nA (Fig. 4K and L). The electrical output of the sensor depended on the actual contact area, leading to the differences in output signals on various surfaces. The as-fabricated sensor exhibited good conformability on a variety of different complex surfaces, confirming its flexibility and excellent shape adaptability and showing great potential for making new green energy wearable sensors.

### Self-powered sensing

**Fig. 5.**
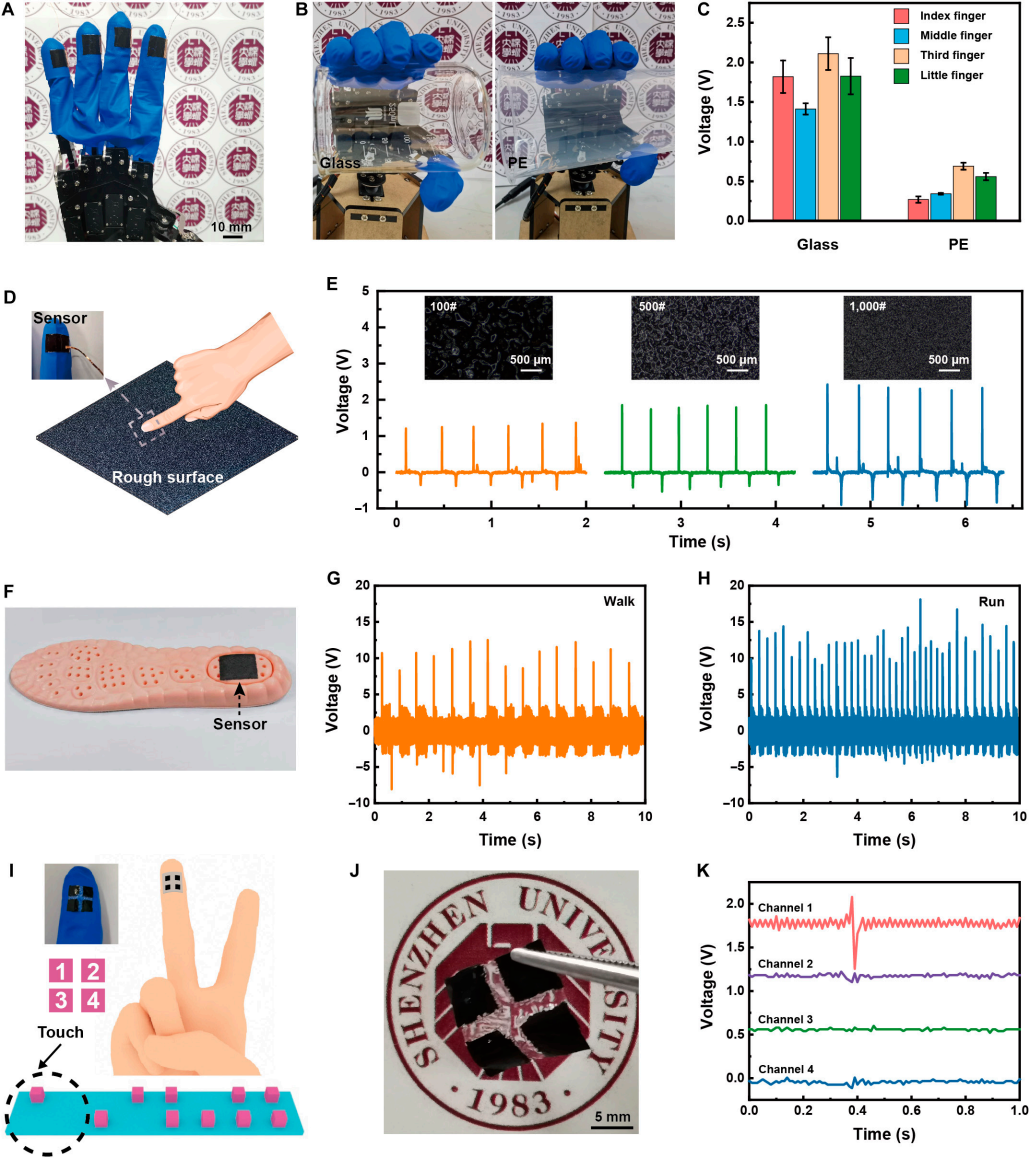
Application of the ultrathin self-powered sensor. (A) Ultrathin sensors attached to the robotic hand. (B) Images and (C) electrical signals of the robotic hand with the sensors when gripping glass and PE. (D) Finger with the ultrathin sensor touching a rough surface. (E) Electrical signals of the sensor touching sandpapers of different meshes with the insert of microscope images of sandpapers. (F) Ultrathin sensor transferred to insole. Electrical signals from the sensor on the insole when (G) walking and (H) running. (I) Diagram of the 2 × 2 sensor array mounted on the index finger. (J) Optical image of the ultrathin sensor array. (K) Electrical signals of the ultrathin sensor array for tactile sensing.

The ultrathin, highly conformal sensor, which operated without an external power source, was capable of being securely affixed to the target body for tactile sensing. The sensors in a size of 10 mm × 10 mm are distributed on each of the 4 fingertips of the robotic hand by the liquid-phase transferring method (Fig. 5A). The 4 sensors were connected to a multi-channel oscilloscope with metal wires to collect voltage signals in real time. The oscilloscope’s negative input was linked to the manipulator’s ground (GND) to minimize electromagnetic interference impact on the sensing signals during manipulation. The voltage signal was selected to characterize the sensing performance, due to its frequency-independent property. When the manipulator grips a glass or polyethylene (PE) cup, the sensors make contact with the objects and can generate corresponding electrical signals (Fig. 5B). When contacting glass, the pulse voltages generated by the 4 sensors on the manipulator are greater than 1.3 V. Next, the manipulator is controlled to grasp a PE cylinder, and the voltage is different from the electrical signal generated by contacting the glass, with a maximum value of no more than 0.7 V (Fig. 5C). The signal peaks that appeared in the 4 fingers when grasping the same material varied slightly due to different strengths. In addition, the polarity difference between 2 contact materials greatly affected the electrical signal output. According to the triboelectric series [45], the difference in electronegativity between glass and PDMS is greater than that between PE and PDMS, and the voltage signals are significantly larger when contacting glass.

The ultrathin self-powered sensor could be utilized to detect roughness. The sensor on the index finger touched the sandpaper with different mesh number sizes (Fig. 5D). The *V*_oc_ signal of 1.2, 2.0, and 2.5 V can be collected when the sensor is in contact with the sandpapers of 100#, 500#, and 1,000#, respectively (Fig. 5E). The lower mesh number represented the higher roughness of the sandpaper. With increased surface roughness, the effective area of contact between the sensor and surface reduced, leading to a decrease in triboelectric charge density and a subsequent reduction in the voltage signal. Touching objects of different roughness resulted in noticeable differences in voltage, which demonstrated the characteristics of roughness recognition. Besides tactile sensing, the sensor (30 mm × 30 mm) could be transferred to an insole to monitor the human motions (Fig. 5F). The *V*_oc_ reaches around 10 V during walking (Fig. 5G) and 14 V during running (Fig. 5H). The variation in *V*_oc_ values across different human motion modes can be attributed to the changes in the contact area between human body and sensor. During running, the feet exert greater force on the device, resulting in an increased contact area and enhancing the *V*_oc_. Additionally, the higher motion frequency associated with running contributes to the observed differences in *V*_oc_ compared to walking.

The ultrathin and flexible sensor array in 2 × 2 pixels was further designed and fabricated, with each pixel in a size of 5 mm × 5 mm (Fig. 5I and J). Through the liquid-phase transferring method, the sensor array could also be attached to the finger. When the finger touched the board with raised dots, electrical signals with differentiated characteristics could be generated by sensing pixels. As shown in Fig. 5K, there is an obvious pulse voltage signal in channel 1, and barely visible signal peaks in channel 2 to channel 4, indicating only one sensing pixel in contact with the raised dot. Based on the triboelectric sensing mode, mechanical stimuli were transformed into electrical signals during the contact and separation process. Therefore, voltage signals were detected at the raised dot positions, and there was no change of electrical signals on the flat surface. Overall, the ultrathin and conformal sensor could be integrated into the target body by liquid-phase transferring method to endow the robot with tactile recognition to realize self-powered sensing, including pressure sensing, material recognition, and roughness detection.

Compared with the previously reported ultrathin triboelectric sensors, the as-fabricated self-powered sensors by liquid-phase transferring exhibited a convenient preparation process, relatively low thickness, superior conformability on multiple complex shaped surfaces, good electrical sensing performances, and abundant application scenarios (Table S1), making them more competitive in wearable devices and human–machine interfacing. Moreover, the presented 45-μm-thick ultrathin multilayer self-powered sensor fabricated via a liquid-phase transferring approach resolves the fundamental trade-off between ultrathin profiles and conformability on complex surfaces in flexible sensing technologies. Unlike conventional wet transfer methods (e.g., PDMS stamp-assisted transfers requiring > 100 μm thickness or 2D material transfers dependent on high-temperature annealing), our innovation employs a water-soluble substrate and spin-coated sandwich architecture, enabling residue-free, mechanical-assistance-free direct transfer through pure water dissolution. This strategy achieves both microscale and macroscale conformability, especially vividly replicating fingerprint-level textures. Leveraging triboelectric effects, the sensor realizes multimodal sensing including pressure, material species, surface roughness, and motion state without external power, demonstrating a great advantage over conventional battery-based sensors. The fabrication process eliminates vacuum deposition and high-temperature treatments, supporting centimeter-scale production, thereby offering a groundbreaking solution for wearable electronics and robotics with integrated ultrathin design, universal conformability, and multifunctional sensing capabilities.

## Discussion

In summary, a liquid-phase transferring method was developed to fabricate the flexible, ultrathin, and highly conformal self-powered sensors in triboelectric mode. The thickness of the entire sensor was 45 μm, which had a sandwich structure with 2 layers of PDMS (triboelectric and supporting layers) and a layer of conductive graphene-containing carbon paste. The sensor could generate electrical signals with the *V*_oc_ of 18.6 V, *I*_sc_ of 1.2 μA, *Q*_sc_ of 22 nC, and a power density of 18 mW m^−2^. The ultrathin sensor demonstrated long-term stability over 4,000 contact–separation cycles. The ultrathin sensor showed outstanding conformability on various complex 3D surfaces, including human skin with fingerprints, robotic hands, insole, flat plates with fine bevels, cylinders, undulating surfaces, and leaf textures. The sensor’s electrical output was influenced by surface morphologies, resulting from the different effective contact areas. As a pressure sensor, the sensitivity was measured at 0.13 V kPa^−1^ within the pressure range of 10 to 40 kPa. The sensor exhibited a rapid response time of 19 ms. Moreover, the sensor and its array could be transferred to the target body, enabling tactile sensing capabilities for pressure, material identification, surface roughness, and motion state detection. This work demonstrated the feasibility of the liquid-phase transferring approach for realizing ultrathin and shape-adaptive self-powered sensors, showing great prospects for applications in wearable technology, robotics, and human–machine interfacing.

## Materials and Methods

### Materials

PDMS (Sylgard 184) was acquired from Dow Corning Inc. Carbon paste was provided by Shanghai Huzheng NANO Technology Co., Ltd. PET films were purchased from Shenzhen Zhongshi Biotechnology Co., Ltd. PVA (95%) is purchased from Macklin. All chemical reagents were used directly without additional purification.

### Preparation of ultrathin sensors by liquid-phase transferring

Firstly, the PET film was cut to a size of 30 mm × 30 mm. PVA solution was spin-coated onto the PET film and then heated in an oven to evaporate the solvent, forming a PVA film layer. The PDMS base and curing agent were combined in a 10:1 ratio. Then, the PDMS precursor was degassed in a vacuum oven and spin-coated on the PVA film, followed by curing at 55 °C for 2 h. Next, the carbon paste was spin-coated on the surface of PDMS and heated at 50 °C for 10 min. Finally, the PDMS precursor was spin-coated onto the top surface and cured at 55 °C for 1 h. The multilayer film was immersed in DI water. After the PVA film was completely dissolved in water, the ultrathin sensor with a 3-layer structure was obtained. When an object’s surface touches the sensor surface in the water, the sensor can be transferred from the water. After evaporating the water, the sensor could be conformably attached to the object.

### Characterizations

The thickness was measured by a Dektak 3ST (Veeco) surface profile testing instrument. The optical microscopic images were recorded using a microscope (Cindbest, CB-M200). Current–voltage curves were performed on a Keithley 2400 source meter. The open-circuit voltage (*V*_oc_), short-circuit current (*I*_sc_), and short-circuit charge (*Q*_sc_) were measured by a LeCroy-62Xs Wave Runner Oscilloscope, a low noise current amplifier (Stanford Research System SR570), and an electrometer (Keithley 6514). The force was measured by a digital force gauge (Sundoo, SH-100).

## Data Availability

The data that support the findings of this study are available in this paper and the Supplementary Materials.

## References

[1] Li H, Tan P, Rao Y, Bhattacharya S, Wang Z, Kim S, Gangopadhyay S, Shi H, Jankovic M, Huh H, et al. E-tattoos: Toward functional but imperceptible interfacing with human skin. Chem Rev. 2024;124(6):3220–3283. 38465831 10.1021/acs.chemrev.3c00626

[2] Qiu Y, Dai X, Yang J, Liao J, Han J, Wu Y, Cao Y, Zhang X, Zhong A, Ni H, et al. An intelligent glove system for real-time multiple electrical signal acquisition and display. Adv Mater Technol. 2024;9(6):2302027.

[3] Tan D, Xu B. Advanced interfacial design for electronic skins with customizable functionalities and wearability. Adv Funct Mater. 2023;33:2306793.

[4] Zheng Q, Dai X, Wu Y, Liang Q, Wu Y, Yang J, Dong B, Gao G, Qin Q, Huang LB. Self-powered high-resolution smart insole system for plantar pressure mapping. BMEMat. 2023;1(1): Article e12008.

[5] Dai X, Wu Y, Liang Q, Yang J, Huang L-B, Kong J, Hao J. Soft robotic-adapted multimodal sensors derived from entirely intrinsic self-healing and stretchable cross-linked networks. Adv Funct Mater. 2023;33(44):2304415.

[6] Nair V, Dalrymple AN, Yu Z, Balakrishnan G, Bettinger CJ, Weber DJ, Yang K, Robinson JT. Miniature battery-free bioelectronics. Science. 2023;382: Article eabn4732. 37943926 10.1126/science.abn4732

[7] Guo ZH, Zhang Z, An K, He T, Sun Z, Pu X, Lee C. A wearable multidimensional motion sensor for AI-enhanced VR sports. Research. 2023;6:0154. 37250953 10.34133/research.0154 PMC10211429

[8] Liao J, Dai X, Han J, Yang J, Wu Y, Cao Y, Qiu Y, Wang Y, Huang L-B, Ni H, et al. Tunable and hierarchically porous self-powered sensor with high sensitivity. Nano Energy. 2024;121:109252.

[9] Zhao H, Shu M, Ai Z, Lou Z, Sou KW, Lu C, Jin Y, Wang Z, Wang J, Wu C, et al. A highly sensitive triboelectric vibration sensor for machinery condition monitoring. Adv Energy Mater. 2022;12(37):2201132.

[10] Dai X, Yang J, Shu C, Liang Q, Han J, Wu Y, Chen M, Cao Y, Ju X, Sun H, et al. Self-powered colorful dynamic electrowetting display systems based on triboelectricity. Small. 2024;20(27):2310359. 10.1002/smll.202310359 38385806

[11] Shi P, Ding Y, Ding B, Xing Q, Kodalle T, Sutter-Fella CM, Yavuz I, Yao C, Fan W, Xu J, et al. Oriented nucleation in formamidinium perovskite for photovoltaics. Nature. 2023;620(7973):323–327. 37344595 10.1038/s41586-023-06208-z

[12] Liu L, Zhang D, Bai P, Mao Y, Li Q, Guo J, Fang Y, Ma R. Strong tough thermogalvanic hydrogel thermocell with extraordinarily high thermoelectric performance. Adv Mater. 2023;35(32):2300696. 10.1002/adma.202300696 37222174

[13] Xu T, Ding X, Cheng H, Han G, Qu L. Moisture-enabled electricity from hygroscopic materials: A new type of clean energy. Adv Mater. 2024;36(12):2209661. 10.1002/adma.202209661 36657097

[14] Shi Y, Shen G. Haptic sensing and feedback techniques toward virtual reality. Research. 2024;7:0333. 38533183 10.34133/research.0333 PMC10964227

[15] Xiang H, Zeng Y, Huang X, Wang N, Cao X, Wang ZL. From triboelectric nanogenerator to multifunctional triboelectric sensors: A chemical perspective toward the Interface optimization and device integration. Small. 2022;18(43): Article e2107222. 36123149 10.1002/smll.202107222

[16] Wang ZL. Triboelectric nanogenerator (TENG)—Sparking an energy and sensor revolution. Adv Energy Mater. 2020;10(17):2000137.

[17] Tao X, Chen X, Wang ZL. Design and synthesis of triboelectric polymers for high performance triboelectric nanogenerators. Energy Environ Sci. 2023;16(9):3654–3678.

[18] Zhang Z, Wen F, Sun Z, Guo X, He T, Lee C. Artificial intelligence-enabled sensing technologies in the 5G/internet of things era: From virtual reality/augmented reality to the digital twin. Adv Intell Syst. 2022;4(7):2100228.

[19] Zheng Q, Zou Y, Zhang Y, Liu Z, Shi B, Wang X, Jin YM, Ouyang H, Li Z, Wang ZL. Biodegradable triboelectric nanogenerator as a life-time designed implantable power source. Sci Adv. 2016;2:2, Article e1501478. 10.1126/sciadv.1501478 PMC4783121 26973876

[20] Tao K, Chen Z, Yu J, Zeng H, Wu J, Wu Z, Jia Q, Li P, Fu Y, Chang H, et al. Ultra-sensitive, deformable, and transparent triboelectric tactile sensor based on micro-pyramid patterned ionic hydrogel for interactive human-machine interfaces. Adv Sci. 2022;9(10):2104168. 10.1002/advs.202104168 PMC8981453 35098703

[21] Chang A, Uy C, Xiao X, Xiao X, Chen J. Self-powered environmental monitoring via a triboelectric nanogenerator. Nano Energy. 2022;98:107282.

[22] Dai X, Huang L-B, Du Y, Han J, Zheng Q, Kong J, Hao J. Self-healing, flexible, and tailorable triboelectric nanogenerators for self-powered sensors based on thermal effect of infrared radiation. Adv Funct Mater. 2020;30(16):1910723.

[23] Wang Z, Jin Y, Lu C, Wang J, Song Z, Yang X, Cao Y, Zi Y, Wang ZL, Ding W. Triboelectric-nanogenerator-enabled mechanical modulation for infrared wireless communications. Energy Environ Sci. 2022;15(7):2983–2991.

[24] Dai X, Huang L-B, Sun Z, Du Y, Xue B, Wong M-C, Han J, Liang Q, Wu Y, Dong B, et al. A phonic braille recognition system based on a self-powered sensor with self-healing ability, temperature resistance, and stretchability. Mater Horiz. 2022;9(10):2603–2612. 35942798 10.1039/d2mh00534d

[25] Wang C, Qu X, Zheng Q, Liu Y, Tan P, Shi B, Ouyang H, Chao S, Zou Y, Zhao C, et al. Stretchable, self-healing, and skin-mounted active sensor for multipoint muscle function assessment. ACS Nano. 2021;15(6):10130–10140. 34086454 10.1021/acsnano.1c02010

[26] Zhang Y, Li Y, Cheng R, Shen S, Yi J, Peng X, Ning C, Dong K, Wang ZL. Underwater monitoring networks based on cable-structured triboelectric nanogenerators. Research. 2022;2022:9809406. 35211679 10.34133/2022/9809406 PMC8837904

[27] Dai X, Liang Q, Zhao Z, Wu Y, Yang J, Han J, Cao Y, Wang Y, Li C-H, Zhong A, et al. Self-powered sensors for flexible electronic skins capable of self-healing under multiple extreme environments. Nano Energy. 2024;121(1):109239.

[28] Pu X, Zhang C, Wang ZL. Triboelectric nanogenerators as wearable power sources and self-powered sensors. Natl Sci Rev. 2023;10(1): Article nwac170. 36684511 10.1093/nsr/nwac170 PMC9843157

[29] Song Z, Yin J, Wang Z, Lu C, Yang Z, Zhao Z, Lin Z, Wang J, Wu C, Cheng J, et al. A flexible triboelectric tactile sensor for simultaneous material and texture recognition. Nano Energy. 2022;93: Article 106798.

[30] Wang H, Han M, Song Y, Zhang H. Design, manufacturing and applications of wearable triboelectric nanogenerators. Nano Energy. 2021;81:105627.

[31] Kang S, Cho S, Shanker R, Lee H, Park J, Um DS, Lee Y, Ko H. Transparent and conductive nanomembranes with orthogonal silver nanowire arrays for skin-attachable loudspeakers and microphones. Sci Adv. 2018;4(8): Article eaas8772. 30083604 10.1126/sciadv.aas8772 PMC6070362

[32] Wu C, Wang AC, Ding W, Guo H, Wang ZL. Triboelectric nanogenerator: A foundation of the energy for the new era. Adv Energy Mater. 2019;9(1):1802906.

[33] Hou K-X, Dai X, Zhao S-P, Huang L-B, Li C-H. A damage-tolerant, self-healing and multifunctional triboelectric nanogenerator. Nano Energy. 2023;116:108739.

[34] Li X, Zhu P, Zhang S, Wang X, Luo X, Leng Z, Zhou H, Pan Z, Mao Y. A self-supporting, conductor-exposing, stretchable, ultrathin, and recyclable kirigami-structured liquid metal paper for multifunctional E-skin. ACS Nano. 2022;16(4):5909–5919. 35312286 10.1021/acsnano.1c11096

[35] Wong TH, Liu Y, Li J, Yao K, Liu S, Yiu CK, Huang X, Wu M, Park W, Zhou J, et al. Triboelectric nanogenerator tattoos enabled by epidermal electronic technologies. Adv Funct Mater. 2022;32:2111269.

[36] Peng X, Dong K, Zhang Y, Wang L, Wei C, Lv T, Wang ZL, Wu Z. Sweat-permeable, biodegradable, transparent and self-powered chitosan-based electronic skin with ultrathin elastic gold nanofibers. Adv Funct Mater. 2022;32(15):2112241.

[37] Cho E, Kim KN, Yong H, Choi WJ, Park J-S, Lee S-J. Highly transparent and water-repellent hierarchical-wrinkled-architecture triboelectric nanogenerator with ultrathin plasma-polymer-fluorocarbon film for artificial triboelectric skin. Nano Energy. 2022;103:107785.

[38] Zhang J-H, Li Z, Xu J, Li J, Yan K, Cheng W, Xin M, Zhu T, Du J, Chen S, et al. Versatile self-assembled electrospun micro-pyramid arrays for high-performance on-skin devices with minimal sensory interference. Nat Commun. 2022;13(1):5839. 36192475 10.1038/s41467-022-33454-y PMC9530173

[39] Jiang Z, Chen N, Yi Z, Zhong J, Zhang F, Ji S, Liao R, Wang Y, Li H, Liu Z, et al. A 1.3-micrometre-thick elastic conductor for seamless on-skin and implantable sensors. Nat Electron. 2022;5(11):784–793.

[40] Zhong Y, Wang J, Wu L, Liu K, Dai S, Hua J, Cheng G, Ding J. Dome-conformal electrode strategy for enhancing the sensitivity of BaTiO_3_-doped flexible self-powered triboelectric pressure sensor. ACS Appl Mater Interfaces. 2023;16(1):1727–1736. 38150505 10.1021/acsami.3c14015

[41] Che L, Hu X, Xu H, Liu Y, Lv C, Kang Z, Wu M, Wen R, Wu H, Cui J, et al. Soap film transfer printing for ultrathin electronics. Small. 2024;20:308312. 10.1002/smll.202308312 37992249

[42] Liang FC, Chang YW, Kuo CC, Cho CJ, Jiang DH, Jhuang FC, Rwei SP, Borsali R. A mechanically robust silver nanowire-polydimethylsiloxane electrode based on facile transfer printing techniques for wearable displays. Nanoscale. 2019;11(4):1520–1530. 30620020 10.1039/c8nr08819e

[43] Le Borgne B, De Sagazan O, Crand S, Jacques E, Harnois M. Conformal electronics wrapped around daily life objects using an original method: Water transfer printing. ACS Appl Mater Interfaces. 2017;9(35):29424–29429. 28831803 10.1021/acsami.7b07327

[44] Wang S, Lin L, Wang ZL. Nanoscale triboelectric-effect-enabled energy conversion for sustainably powering portable electronics. Nano Lett. 2012;12(12):6339–6346. 23130843 10.1021/nl303573d

[45] Khandelwal G, Raj N, Kim SJ. Materials beyond conventional triboelectric series for fabrication and applications of triboelectric nanogenerators. Adv Energy Mater. 2021;11(33):2101170.

