# Supplementary material for: Ultrathin and Highly Conformal Self-Powered Sensors by Liquid-Phase Transferring
Source: Research (Wash D C). 2025 Jul 29;8:0785. doi: 10.34133/research.0785 (PMC12304740; doi:10.34133/research.0785)
Supplement: Supplementary 1 — Table S1 [file research.0785.f1.docx]

**Supplementary Information**

**Ultrathin and Highly Conformal Self-Powered Sensors by Liquid-Phase Transferring**

Xingyi Dai^1, 2^, Qihua Liang^1^, Yinghui Wu^1^, Jiaxin Han^1^, Yajun Cao^1^, Xuyang Zhang^1^, Junhui Huang^1^, Junle Qu^1^, Long-Biao Huang^1, 4,^*, Jie Kong^3,^ *, Jianhua Hao^2,^ *

^1^ Key Laboratory of Optoelectronic Devices and Systems of Ministry of Education and Guangdong Province, College of Physics and Optoelectronic Engineering, Shenzhen University, Shenzhen, 518060, P. R. China

^2^ Department of Applied Physics, The Hong Kong Polytechnic University, Hong Kong, P. R. China

^3^ MOE Key Laboratory of Materials Physics and Chemistry in Extraordinary Conditions, Shaanxi Key Laboratory of Macromolecular Science and Technology, School of Chemistry and Chemical Engineering, Northwestern Polytechnical University, Xi’an, 710072, P. R. China.

^4^ National Key Laboratory of Green and Long-Life Road Engineering in Extreme Environment, Shenzhen University, Shenzhen 518060, P. R. China

*Address correspondence to: [huanglb@szu.edu.cn](mailto:huanglb@szu.edu.cn) (Long-Biao Huang); [jh.hao@polyu.edu.hk](mailto:jh.hao@polyu.edu.hk) (Jianhua Hao); and [kongjie@nwpu.edu.cn](mailto:kongjie@nwpu.edu.cn) (Jie Kong)

**Table S1.** Summary of the performances of various ultrathin triboelectric sensors

| **Material** | **Fabrication method** | **Thickness** | **Conformability** | **Power density** | **Sensitivity** | **Application** | **Ref.** |
| --- | --- | --- | --- | --- | --- | --- | --- |
| PDMS, BaTiO_3_/PDMS, AgNWs | Spin coating | > 50μm (13.2 μm of outermost  PDMS) | Fixed on the  Human body with a cling film | 17.68 mW m^−2^ | 0.75 V/kPa (0−26 kPa), 0.19 V/kPa (26−120 kPa) | Pressure detection | [1] |
| Cu/PI/PDMS | spin coating, sputtering, and photolithography | ~ 50 μm | Mounted on a human skin surface by liquid bandage | ~ 21.12 mW cm^−3^ | – | Human  machine  interface,  energy  harvester | [2] |
| CNF/PVA/LM | Casting | < 50 μm | Put on the human body | 88 mW m^−2^ | – | E-skin | [3] |
| PPFC/SEBS/  Ag-SEBS, Al | Blade coating and sputtering | PPFC: 0.1 μm, SEBS: 20 μm, Al: 50 μm | Attached to human body | 11 W m^−2^ | – | Raindrops energy harvester and touch skin | [4] |
| Electrospun fibers, Au-coated fibers | Electrospinning | > 50 μm | laminated on the fingertip with electrospun PVA nanofiber glue | – | – | Pressure sensing, and bioenergy  harvesting | [5] |
| CS/Au NFs/CS | Solution-casting, electrospinning, magnetron sputtering, and photolithography | 10 μm | Placed on the skin surface | – | 0.012 kPa^−1^ (0−70 kPa) | E-skin | [6] |
| P(VDF-TrFE)  /PDMS/Ag | Solvent-assisted micromolding | Below ∼400 μm | Worn on the human body | 46.7 μW  cm^−2^ | 0.55 V kPa^−1^ | Detecting pressing, bending, and  twisting motions | [7] |
| PDMS/ITO-PET | Spin coating | 92 μm | Attached to an acrylic plate with double sided adhesive | – | – | Real-time alarming and accurate positioning | [8] |
| CA-M NFs, CA-NH_2_ NFs, bead PVDF NFs, and carbon NFs | electrospinning | 200 μm | Attached to the skin | 81 mW m^−2^ | 0.32 V kPa^−1^ (0−135 kPa) | Monitoring of sleep quality | [9] |
| PDMS, TPU/AgNWs, TPU, VHB tap | electrospinning | 89 μm | Attached on human skin | 6 mW m^−2^ | 9.973 mV Pa^−1^ (0−1.6 kPa), 0.538 mV Pa^−1^ (1.6−7 kPa) | Haptic sensor for amusement games | [10] |
| PDMS, graphene-containing carbon paste | Spin-coating and liquid-phase transferring techniques | 45 μm | Attached on human skin, manipulator hand, insole, flat plate with fine bevel, cylinder surface, undulating surface, and leaf textures | 18 mW m^−2^ | 0.13 V kPa^−1^ (10−40 kPa) | Tactile sensing for detecting pressure, material species, roughness, and motion state | This work |

PDMS – polydimethylsiloxane, AgNWs – silver nanowires, PI – polyimide, CNF – cellulose nanofibril, PVA – poly(vinyl alcohol), LM – liquid metal, PPFC – plasma-polymer-fluorocarbon, SEBS – styrene-ethylene-butylene-styrene, CS – chitosan, Au NFs – gold nanofibers, P(VDF-TrFE) – poly(vinylidenefluoride-co-trifluoroethylene), ITO-PET – indium tin oxide-polyethylene terephthalate, CA – collagen aggregate, CA-M NFs – CA nanofibers doped with acidified carbon nanotubes, CA-NH_2_ NFs – CAs modified with KH-792 nanofibers, bead PVDF NFs – polyvinylidene fluoride nanofibers designed with a delicate bead chain structure, TPU – thermoplastic polyurethane.

**References**

1. Zhong Y, Wang JQ, Wu LG, et al. Dome-Conformal Electrode Strategy for Enhancing the Sensitivity of BaTiO-Doped Flexible Self-powered Triboelectric Pressure Sensor. *ACS Appl Mater Interfaces*. 2023;16:1727-1736.

2. Wong TH, Liu Y, Li J, et al. Triboelectric Nanogenerator Tattoos Enabled by Epidermal Electronic Technologies. *Adv Funct Mater*. 2022;32:2111269.

3. Li X, Zhu PC, Zhang SC, et al. A Self-Supporting, Conductor-Exposing, Stretchable, Ultrathin, and Recyclable Kirigami-Structured Liquid Metal Paper for Multifunctional E-Skin. *ACS Nano*. 2022;16:5909-5919.

4. Cho EM, Kim KN, Yong H, Choi WJ, Park JS, Lee SJ. Highly transparent and water-repellent hierarchical-wrinkled-architecture triboelectric nanogenerator with ultrathin plasma-polymer-fluorocarbon film for artificial triboelectric skin. *Nano Energy*. 2022;103:107785.

5. Zhang JH, Li ZT, Xu J, et al. Versatile self-assembled electrospun micro-pyramid arrays for high-performance on-skin devices with minimal sensory interference. *Nat Commun*. 2022;13:5839.

6. Peng X, Dong K, Zhang YF, et al. Sweat-Permeable, Biodegradable, Transparent and Self-powered Chitosan-Based Electronic Skin with Ultrathin Elastic Gold Nanofibers. *Adv Funct Mater*. 2022;32:2112241.

7. Ha M, Lim S, Cho S, et al. Skin-Inspired Hierarchical Polymer Architectures with Gradient Stiffness for Spacer-Free, Ultrathin, and Highly Sensitive Triboelectric Sensors. *ACS Nano*. 2018;12:3964-3974.

8. Wang BC, Zhai XY, Wei XL, et al. A self-powered and concealed sensor based on triboelectric nanogenerators for cultural-relic anti-theft systems. *Nano Research*. 2022;15:8435-8441.

9. Yue OY, Wang XC, Hou MD, et al. Skin-inspired wearable self-powered electronic skin with tunable sensitivity for real-time monitoring of sleep quality. *Nano Energy*. 2022;91:106682.

10. Jiang Y, Dong K, Li X, et al. Stretchable, Washable, and Ultrathin Triboelectric Nanogenerators as Skin-Like Highly Sensitive Self-Powered Haptic Sensors. *Adv Funct Mater*. 2020;31:2005584.
